# The protective effects of Mirtazapine against lipopolysaccharide (LPS)-induced brain vascular hyperpermeability

**DOI:** 10.1080/21655979.2021.2024962

**Published:** 2022-01-26

**Authors:** Yuehong Pu, Lei Zhao, Yao Xi, Yichun Xia, Yiming Qian

**Affiliations:** Department of Emergency Medicine, Yueyang Hospital of Intergrated Traditional Chinese and Western Medicine, Shanghai University of Traditional Chinese Medicine, Shanghai, China

**Keywords:** Sepsis, Mirtazapine, blood-brain barrier, ZO-1, Nrf2

## Abstract

Sepsis is mainly characterized by severe inflammation triggered by infection, and sepsis-associated encephalopathy (SAE) is defined as brain damage caused by sepsis. Disruption of the blood-brain barrier (BBB) triggered by injured brain microvascular endothelial cells (BMECs) and damaged tight junction (TJ) structure is closely associated with the pathogenesis of SAE. The present research proposed to evaluate the potential therapeutic effects of Mirtazapine, a central presynaptic α2 receptor antagonist, on LPS-induced BBB disruption. The mice were administered with normal saline and 10 mg/kg Mirtazapine for 8 consecutive days, and from day 6, the experiment group of mice received LPS for 2 days to induce SAE. We found that the increased BBB permeability, elevated concentrations of inflammatory factors in brain tissues, and downregulated zonula occludens −1 (ZO-1) were observed in LPS-stimulated mice, all of which were reversed by 10 mg/kg Mirtazapine. In the *in vitro* assay, bEnd.3 brain endothelial cells were treated with 1 μM LPS in the absence or presence of Mirtazapine (25, 50 μM). We found that LPS-treated cells had significantly declined transendothelial electrical resistance (TEER), increased monolayer permeability, elevated production of inflammatory factors, and downregulated ZO-1. However, 25 and 50 μM Mirtazapine ameliorated all these LPS- induced aberrations. Mirtazapine also mitigated the decreased level of NF-E2–related factor 2 (Nrf2) in LPS-challenged endothelial cells. The protective effect of Mirtazapine on endothelial permeability against LPS was significantly abolished by the knockdown of Nrf2. Collectively, we concluded that Mirtazapine exerted protective effects on LPS-induced endothelial cells hyperpermeability by upregulating Nrf2.

## GRAPHICAL ABSTRACT

**Figure:**
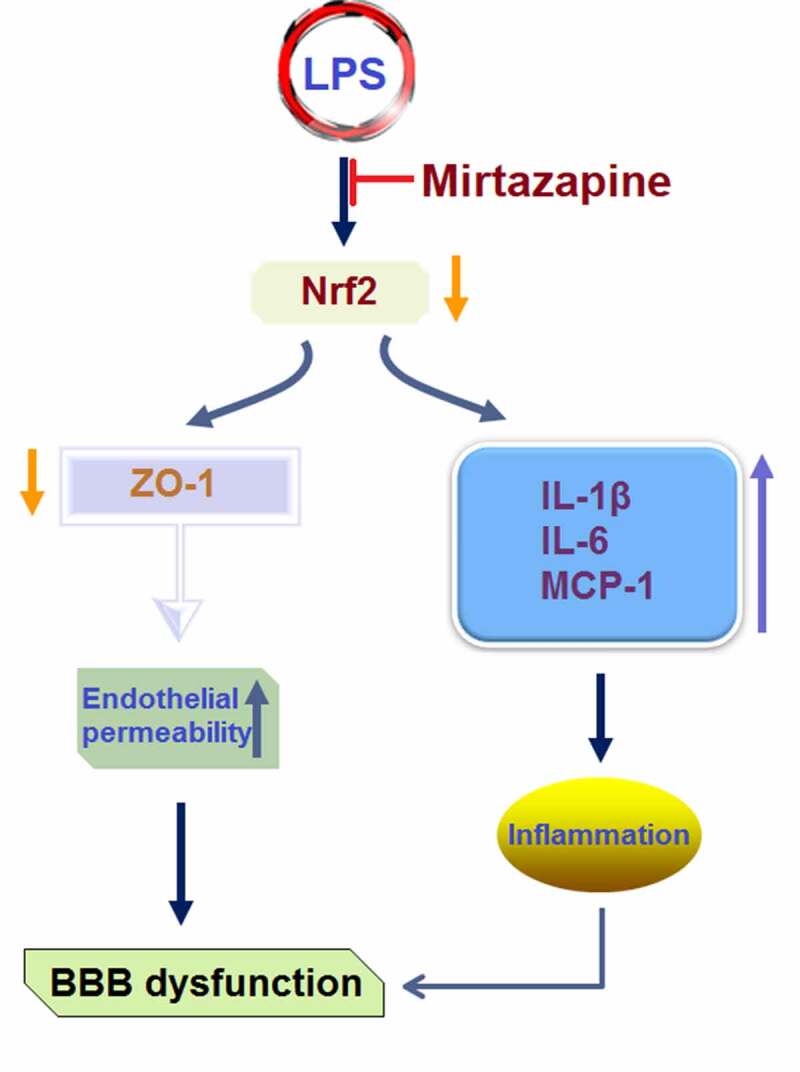

## Introduction

Sepsis is an infection-triggered systemic inflammatory reaction, the development of which induces acute dysfunction on multiple organs [1,2]. Sepsis-associated encephalopathy (SAE) is a type of sepsis-triggered brain damage characterized by symptoms of cognitive dysfunction, with morbidity ranging from 7% to 81% [3]. It is mainly triggered by disorders of brain microcirculation, nerve injury, and disruption of the blood-brain barrier (BBB) [4–6]. By separating the circulating blood system from brain tissue, the BBB exerts a critical role in mediating the steady state of the brain to keep normal function. It mainly consists of BMECs, TJs, and astrocyte foot processes [7,8]. Neuroinflammation is induced in brain tissues by signals delivered from the peripheral system via the vagus nerve. The microglia are activated to release inflammatory mediators, such as TNF-α, IL-1β, and prostaglandin, contributing to BBB damage together with bacteria, LPS, complements, cytokines, and chemokines. As a consequence, severe overactivation and injuries are induced on BMECs, intumescent astrocytes and pericytes are generated, and the BBB permeability is increased to contribute to its dysfunction [9]. Dramatically promoted protein concentration is reported to be observed in the cerebrospinal fluid (CSF) of SAE patients [10]. Edema was detected in brain tissues by magnetic resonance imaging (MRI) [11]. The energetic dysfunction in BMECs is induced by the ischemic and hypoxia states during sepsis. The interaction between zona occludens 1 (ZO-1) and filamentous actin (F-actin) is enhanced to promote the calcium influx and activate multiple transcriptional factors, which further downregulate TJ proteins and trigger BBB disruption [12]. The balance between pro-inflammation and anti-inflammation is disrupted by the inflammatory factors and chemokines released by damaged BMECs. The pro-inflammatory elements in the circulation are then introduced into the CNS through the disrupted BBB, activating the astrocytes and microglia [13] to aggravate the neuroinflammation and mediate the oxidative stress and mitochondrial dysfunction [14]. Therefore, protecting BBB permeability is an effective strategy for treating SAE.

Mirtazapine is a central presynaptic α2 receptor antagonist which regulates the function of serotonin (5-HT) and enhances the adrenergic nerve transduction by interacting with the 5-HT receptors [15]. It shows effective therapeutic properties on post-traumatic stress disorder, primary anxiety disorder, central post-stroke pain, and multiple types of depression [16]. Recently, it has been reported that Mirtazapine exerts promising anti-inflammatory effects in diabetic rats [17] and ulcerative colitis (UC) [18].

Neuroinflammation and ischemic injury are primary pathological characteristics of SAE. Since Mirtazapine exhibits potent anti-inflammatory properties and improves neurovascular conditions, we hypothesized that it could be potentially tested in experimental SAE animals. In rodents, peripheral administration of the bacterial endotoxin lipopolysaccharide (LPS) to induce SAE has been widely used as a convenient tool to assess inflammation and neurovascular impairment [19]. In this study, we aimed to evaluate the potential therapeutic effect of Mirtazapine and the molecular mechanism in LPS-induced SAE mice.

## Materials and methods

### Animal experiments

Male 6-to 8-week old male C57BL/6 mice were purchased from the Laboratory Animal Center of Peking University. The mice were housed in a 12 hour light/dark cycle facility with a constant temperature (22°C), with food and water ad libitum. Lipopolysaccharide (LPS) was obtained from Sigma-Aldrich (# L2880). Mice were randomly divided into 4 groups: Sham, Mirtazapine, LPS, and LPS+ Mirtazapine groups. The mice were pretreated with Mirtazapine for 6 days and then were given LPS to induce SAE for 48 hours. During the sepsis induction period, the mice also received Mirtazapine treatment. In brief, mice in the Sham group were administered intraperitoneally with sterile 0.9% normal saline, and the mice in the Mirtazapine group were only treated intraperitoneally with 10 mg/kg Mirtazapine once a day for 8 consecutive days. Mice in the LPS group were only administered intraperitoneally with 3 mg/kg of LPS once on the 6^th^ day. For the LPS+ Mirtazapine group, the mice were treated with Mirtazapine (intraperitoneal (i.p.), 10 mg/kg) once a day for 8 consecutive days and received LPS (i.p. 3 mg/kg) on the 6^th^ day as described previously [20]. The animals were sacrificed after 8-day Mirtazapine treatment (i.e. two days after LPS injection). Experimental protocols were approved by the Ethical Committee of Yueyang Hospital of Integrated Traditional Chinese and Western Medicine.

### Measurement of brain/serum weight ratio and brain endothelial permeability

Mice were injected with ^14^C sucrose (i.v., 10^6^ dpm in 0.2 ml of lactated Ringer’s solution with 1% BSA) 24 hours after LPS administration as described in a previous study. 24 hours after sucrose labeling, the mice were euthanized by CO_2_ inhalation [21]. The blood samples were collected from the descending abdominal aorta. To perfuse the tissue, the thorax was opened, and the descending abdominal aorta was clamped. The jugular veins were severed, and the lactated Ringer’s Solution was perfused through the left ventricle to remove the blood from the brain tissues. The brain tissues were dissected and weighed, then centrifuged at 4000 × g for 10 minutes, and the serum was obtained. A beta counter was utilized to measure the concentration of radioactivity in serum and brain tissues to determine the concentration of ^14^C sucrose. The permeability of the BBB was calculated by dividing the concentration of ^14^C sucrose in the serum by the concentration of ^14^C sucrose in brain tissues.

### Real-time PCR analysis

The isolation of total RNAs from cells and tissues was conducted using the TRIZOL reagent (Invitrogen, California, USA). The RNA concentration was measured using a Nanodrop machine (Themo Fisher Scientific, USA). Total 2 µg of pure RNA was reversely transcribed into cDNAs utilizing the TaqMan reverse transcription kit (Invitrogen. California, USA). The ABI 7900 real-time PCR machine was applied to conduct the PCR reaction with SYBR Green Real‐time PCR Master Mix (Roche Diagnostics, Basel, Switzerland). GAPDH was used for the normalization of expression of genes, which was determined using the 2^−ΔΔCt^ method [22]. The PCR cycle consists of 40 cycles with three steps: denature step at 95°C /3 seconds, annealing step at 60°C/20 seconds, extension step at 72°C /20 seconds. The following primers were used: Mu ZO-1: F: 5’-GTTGGTACGGTGCCCTGAAAGA-3’, R: 5’-GCTGACAGGTAGGACAGACGAT-3’; Mu IL-1β: F: 5’-TGGACCTTCCAGGATGAGGACA-3’, R:5’-GTTCATCTCGGAGCCTGTAGTG-3’; Mu IL-6: F: 5’-TACCACTTCACAAGTCGGAGGC-3’, R: 5’-CTGCAAGTGCATCATCGTTGTTC-3’; Mu MCP-1: F: 5’-GCTACAAGAGGATCACCAGCAG-3’, R: 5’-GTCTGGACCCATTCCTTCTTGG-3’; Mu GAPDH: F: 5’-CATCACTGCCACCCAGAAGACTG-3’, R: 5’- ATGCCAGTGAGCTTCCCGTTCAG-3’; Hu IL-1β: F: 5’- CCACAGACCTTCCAGGAGAATG-3’, R: 5’- GTGCAGTTCAGTGATCGTACAGG-3’; Hu IL-6: F: 5’- AGACAGCCACTCACCTCTTCAG-3’, R: 5’-TTCTGCCAGTGCCTCTTTGCTG-3’; Hu MCP-1: F: 5’- AGAATCACCAGCAGCAAGTGTCC-3’, R: 5’-TCCTGAACCCACTTCTGCTTGG-3’; Hu ZO-1: F: 5’- GTCCAGAATCTCGGAAAAGTGCC-3’, R: 5’-CTTTCAGCGCACCATACCAACC-3’; Hu Nrf2: F: 5’- CACATCCAGTCAGAAACCAGTGG-3’, R: 5’- GGAATGTCTGCGCCAAAAGCTG-3’; GAPDH: F: 5’- GTCTCCTCTGACTTCAACAGCG-3’, R: 5’-ACCACCCTGTTGCTGTAGCCAA-3’.

### Immunostaining

In brief, brain tissues were fixed in 4% paraformaldehyde, dehydrated, paraffin embedded, and sectioned. After being baked and dehydrated, slides were incubated with 5% skim milk, followed by adding the primary antibody against ZO-1 (1:500, R&D, Minnesota, USA). To ensure specificity, two negative control experiments were performed without primary antibody or secondary antibody. After three washes, slides were incubated with the Alexa Fluor 594 goat anti-rabbit antibody solution (R&D, Minnesota, USA) at 37°C for 45 minutes. Slides were checked under a fluorescence microscope (KEYENCE, Tokyo, Japan).

### Western blot assay

Following the extraction of proteins from tissues and cells, the proteins were quantified with a BCA protein assay kit (Thermo Scientific Inc. USA). The total 20 µg protein samples were loaded onto the 12% SDS-PAGE. After electrophoresis for 1.5 hours, proteins were transferred onto the PVDF membrane (Takara, Tokyo, Japan) and blocked using the 5% skim milk, followed by adding the primary antibody against ZO-1 (1:1000, Affintiy, Melbourne, Australian), Nrf2 (1:1000, Affintiy, Melbourne, Australian), and β-actin (1:1000, Affintiy, Melbourne, Australian), respectively. After incubation with the secondary antibody (1:2000, Affintiy, Melbourne, Australian) for 1.5 hours, the membrane was reacted with the ECL solution. To quantify the result, the Western blot bands on the films were scanned. The total optical density of target bands was calculated with Image J software (NIH, USA). Bands of target proteins were selected and the background was subtracted. The quantitated results were then exported for statistical analysis. Expression of the target proteins was normalized to β-actin.

### ELISA assay

The secretion of IL-1β, IL-6, and MCP-1 was evaluated using the ELISA assay (CST, California, USA) 48 hours post LPS injection. Briefly, after seeding samples and standards onto the 96-well plate, samples were incubated for 60 minutes at 37°C, followed by removing the culturing medium and adding the conjugate solution to be incubated for 30 minutes. Then, the TMB solution was added for 15 minutes incubation followed by adding the stop solution. Finally, the absorbance at 450 nm was checked using a microplate reader (Bruker, Massachusetts, USA) for the calculation of the concentration of the proteins, and the unit was shown in pg/mg tissue or pg/mL cellular protein.

### Cell culture and treatment, and ad-viral Nrf2 shRNA transduction

Mouse bEnd.3 brain endothelial cells were obtained from ATCC (California, USA) and cultured in the DMEM containing 4.5 mg/mL glucose and 10% FBS at 37°C and 5% CO_2._ To knockdown the expression level of Nrf2, cells were transfected with 10 multiplicity of infection (MOI) of the ad-viral Nrf2 shRNA or scramble shRNA (Genscript, Nanjing, China) together with the lip3000 (100,000 cells/µl, Life technologies) for 2 days. The efficacy of transfection was identified with the Western blotting assay.

### Transendothelial electrical resistance (TEER)

The endothelial permeability was detected using a TEER assay with a 1600 R ECIS System (Applied Biophysics, Sydney, Australia) according to the method described [23]. An average of the resistance values (Ω·cm^2^) and the average percent change from baseline TEER (Mean ± SD) were used to express the results.

### Endothelial permeability was measured using FITC-dextran

After plating cells onto the luminal side of filters (0.4 μM pore size, Millipore, Massachusetts, USA) and incubating overnight, the cells were treated with LPS (1 μM) in the presence or absence of Mirtazapine (50 μM) for 24 hours. To test the effect of Nrf2, the cells were infected with ad-viral Nrf2 shRNA or scramble shRNA for 24 hours, then treated with LPS and Mirtazapine. Afterward, 1 mg/mL FITC-dextran 40 (40 KDa) (MedChem Express, New Jersey, USA) was introduced into the upper chamber to be incubated for 60 minutes, followed by collecting the sample in the lower chamber. A microplate reader (TECAN, Männedorf, Switzerland) was utilized to determine the absorbance at 492/520 nm.

### Statistical analysis

Data obtained in the present study were expressed as mean ± standard deviation (S.D.). The results were analyzed using the GraphPad software Prism 6.0. The Student’s t-test was utilized to analyze the difference between the 2 groups and the one-way ANOVA method followed by Tukey’s post-hoc test was applied to analyze the differences among groups. P < 0.05 was regarded as a significant difference in the present study.

## Results

Our data shows that Mirtazapine therapy significantly improved SAE- associated brain vascular permeability and inflammation. In *in vitro* cultured brain endothelial cells, Mirtazapine treatment ameliorated LPS- induced endothelial hyperpermeability and inflammation. Using a knockdown experiment, we found that Nrf2 is a critical factor involved in the effect of Mirtazapine.

### Mirtazapine attenuates LPS treatment-induced BBB disruption

**Figure 1.**
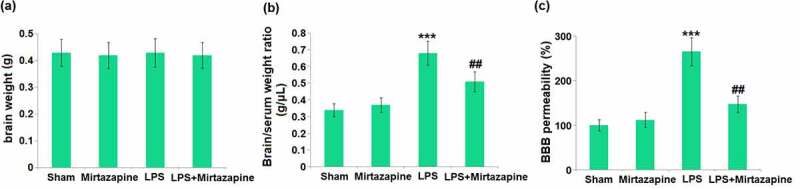
Mirtazapine attenuates LPS treatment-induced BBB disruption. The mice were weighed at day 6 before the LPS injection and weighed again at day 7, about 24 hours after the LPS injection. (a) Change in brain weight; (b) Brain/Serum weight ratio; (c) The BBB permeability to ^14^C-Sucrose (***, P < 0.005 vs. LPS sham group; ##, P < 0.01 vs. LPS group).

Mice were weighed on day 6 before the LPS injection and weighed again on day 8, about 48 hours after the LPS injection, followed by measuring the brain weights and determining the BBB permeability. No significant difference was observed in brain weights amongst the 4 groups (Figure 1(a)). The brain/serum ratio (Figure 1(b)) was indistinctively changed from 0.34 g/μL to 0.37 g/μL in the Mirtazapine group and significantly promoted to 0.68 g/μL by LPS. It was then repressed to 0.51 g/μL by the co-administration of Mirtazapine. Additionally, the permeability to ^14^C-Sucrose (Figure 1(c)) in the Sham, Mirtazapine, LPS, and LPS+ Mirtazapine groups was 100%, 112.2%, 265.4%, and 147.6%, respectively. These data collectively reveal that the BBB disruption in LPS-treated mice was alleviated by Mirtazapine.

### Mirtazapine attenuated LPS treatment-induced elevation of cytokines in the brain

**Figure 2.**
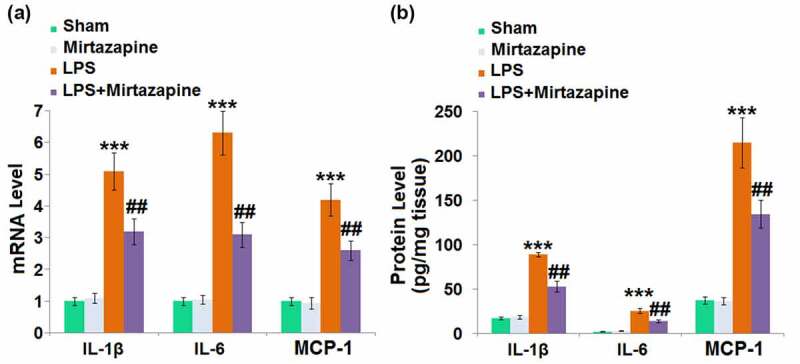
Mirtazapine attenuates LPS treatment-induced elevation of cytokines in the brain. (a) mRNA level of IL-1β, IL-6, MCP-1; (b) Protein levels of IL-1β, IL-6, MCP-1 was determined (***, P < 0.005 vs. LPS sham group; ##, P < 0.01 vs. LPS group).

Severe inflammation is the main characteristic of SAE [24]. We further measured the production of inflammatory factors in brain tissues. The expression levels of IL-1β, IL-6, and MCP-1 (Figure 2(a)) were slightly changed in the Mirtazapine group and dramatically promoted in the LPS group. They were greatly reduced in the LPS+ Mirtazapine group. The protein levels of IL-1β (Figure 2(b)) in the Sham, Mirtazapine, LPS, and LPS+ Mirtazapine groups were 17.4, 18.7, 89.3, and 53.1 pg/mg tissue, respectively. The release of IL-6 was indistinctively switched from 2.4 pg/mg tissue to 3.1 pg/mg tissue after the treatment with Mirtazapine in Sham animals and significantly promoted to 25.7 pg/mg tissue by the injection of LPS. It was then greatly decreased to 14.5 pg/mg tissue in the LPS+ Mirtazapine group. Lastly, the protein levels of MCP-1 in the Sham, Mirtazapine, LPS, and LPS+ Mirtazapine group were 37.6, 36.8, 214.7, and 134.6 pg/mg tissue, respectively. These data collectively reveal that the severe inflammation in brain tissues triggered by LPS was pronouncedly mitigated by Mirtazapine.

### Mirtazapine elevated the ZO-1 level inhibited by LPS treatment in the brain

**Figure 3.**
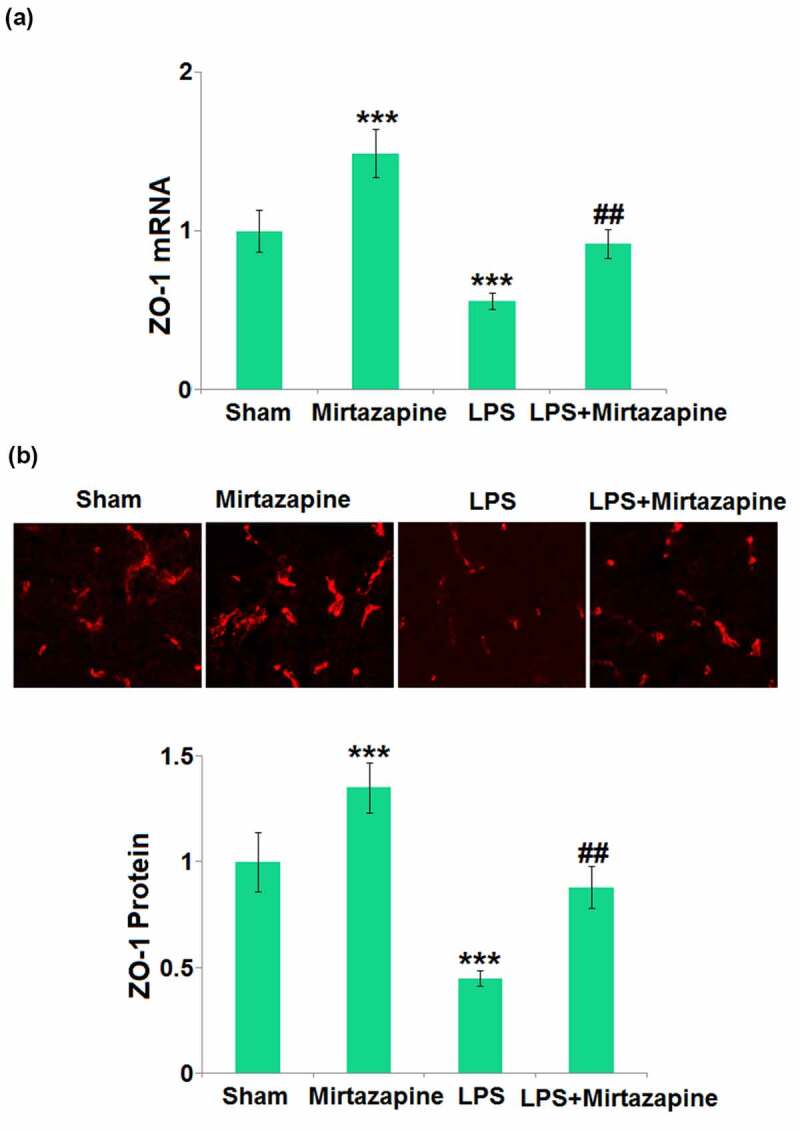
Mirtazapine elevates the expression of ZO-1 inhibited by LPS treatment in the brain. (a) mRNA level of ZO-1; (b) immunostaining of ZO-1, the representative images from each group were shown in the upper panel, the quantitative plot was shown in the lower panel (***, P < 0.005 vs. LPS sham group; ##, P < 0.01 vs. LPS group).

ZO-1 is critical for the integrity of TJs and the BBB [24]. The ZO-1 (Figure 3(a,b)) level in the Mirtazapine group was significantly higher than that in the Sham group. After stimulation with LPS, ZO-1 in the brain tissue was dramatically downregulated. Similarly, the ZO-1 level in the LPS+ Mirtazapine group was dramatically higher than that in the LPS group, indicating that ZO-1 might be an important mediator responsible for the protective effect of Mirtazapine on BBB integrity.

### Impacts of Mirtazapine on LPS treatment-challenged cell permeability in Bend.3 brain endothelial cells

**Figure 4.**
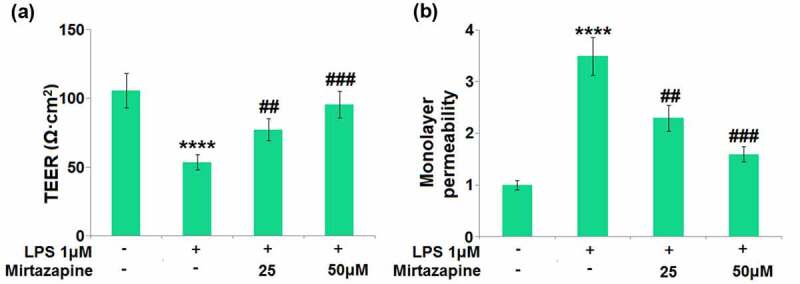
Effect of Mirtazapine on LPS treatment-induced cell permeability in Bend.3 brain endothelial cells. Cells were treated with LPS (1 μM) in the absence or presence of Mirtazapine (25, 50 μM). (a). TEER on the endothelial monolayer was measured; (b) Monolayer permeability was measured using FITC-dextran permeability assay (****, P < 0.001 vs. normal mice group; ##, ###, P < 0.01, 0.005 vs. LPS treatment group).

BMECs are important components of the BBB reported to regulate the permeability of brain endothelial function [25]. We found that the TEER on the endothelial monolayer (Figure 4(a)) was significantly decreased from 105.8 Ω·cm^2^ to 53.6 Ω·cm^2^ in LPS-treated brain endothelial cells, then greatly promoted to 77.3 and 95.6 Ω·cm^2^ by 25 and 50 μM Mirtazapine, respectively. The results of FITC-dextran (Figure 4(b)) indicate that the monolayer permeability increased in LPS-treated brain endothelial cells was greatly repressed by 25 and 50 μM Mirtazapine. These data collectively reveal that the increased endothelial permeability was mitigated by Mirtazapine.

### Effects of Mirtazapine on LPS treatment-induced cytokines in Bend.3 brain endothelial cells

**Figure 5.**
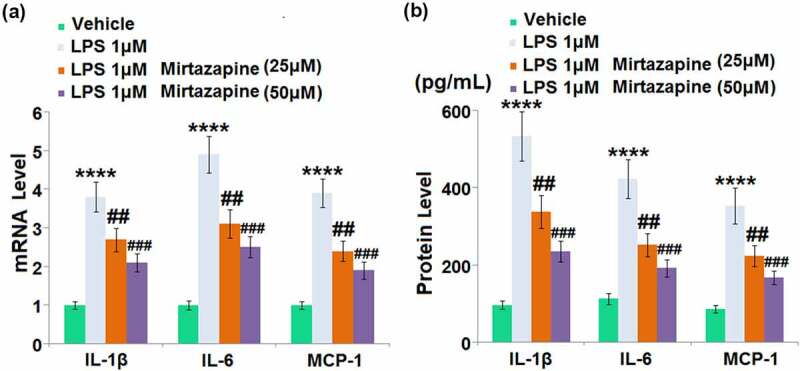
Effect of Mirtazapine on LPS treatment-induced cytokines in Bend.3 brain endothelial cells. Cells were treated with LPS 1 μM in the absence or presence of Mirtazapine (25, 50 μM). (a). mRNA level of IL-1β, IL-6, MCP-1 was determined; (b). Concentrations of IL-1β, IL-6, and MCP-1 (****, P < 0.001 vs. normal mice group; ##, ###, P < 0.01, 0.005 vs. LPS treatment group).

We further checked the impact of Mirtazapine on the production of inflammatory factors in LPS-treated endothelial cells. We treated bEnd.3 cells with LPS for 48 hours in the presence or absence of Mirtazapine. Upregulated gene expressions of IL-1β, IL-6, and MCP-1 (Figure 5(a)) were found in LPS-treated endothelial cells, all of which were greatly downregulated by 25 and 50 μM Mirtazapine. The release of IL-1β (Figure 5(b)) was dramatically enhanced from 96.5 pg/mL to 533.1 pg/mL by LPS, then greatly declined to 337.2 and 235.5 pg/mL by 25 and 50 μM Mirtazapine, respectively. The production of IL-6 in the control, LPS, 25 Mirtazapine, and 50 μM Mirtazapine groups was 112.7, 423.5, 252.1, and 191.9 pg/mL, respectively. Additionally, the release of MCP-1 in LPS-treated cells was increased from 86.2 pg/mL to 352.9 pg/mL, which was greatly declined to 224.1 and 167.5 pg/mL in the 25 Mirtazapine and 50 μM Mirtazapine groups, respectively. These results collectively indicate that the severe inflammation facilitated by LPS was greatly improved by Mirtazapine.

### Mirtazapine reserved the expression of tight junction proteins inhibited by LPS treatment

**Figure 6.**
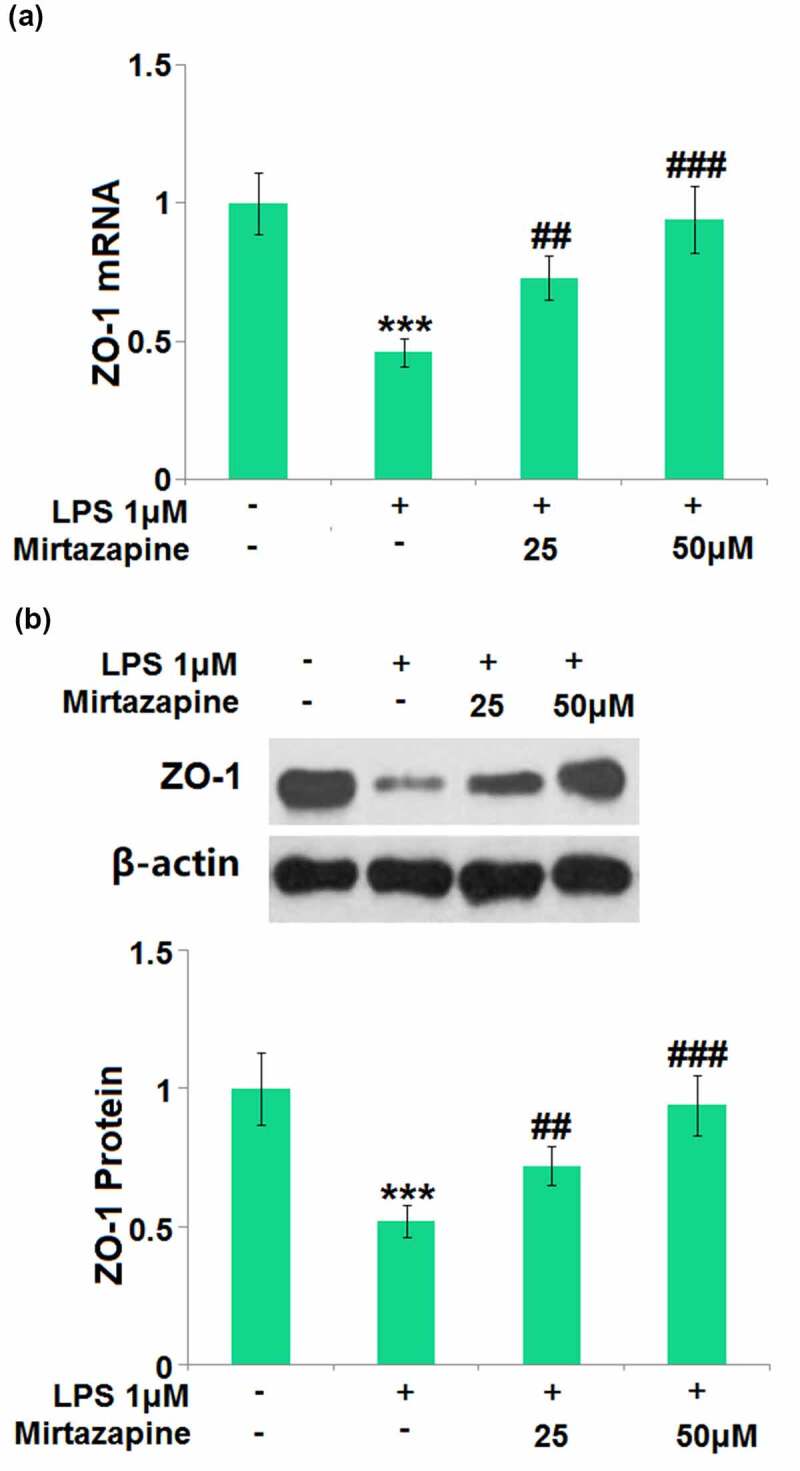
Mirtazapine reserved the expression of tight junction proteins inhibited by LPS treatment in Bend.6. Cells were treated with LPS 1 μM in the presence or absence of Mirtazapine (25, 50 μM). (a). mRNA of ZO-1; (b). Protein expression of ZO-1. The representative blots of ZO-1 and β-actin were shown in the upper panel, the quantitative plot was shown in the lower panel (****, P < 0.001 vs. normal mice group; ##, ###, P < 0.01, 0.005 vs. LPS treatment group).

The impact of Mirtazapine on the ZO-1 level in LPS-treated endothelial cells was further checked. ZO-1 (Figure 6(a,b)) was found significantly downregulated by the stimulation with LPS but greatly upregulated by 25 Mirtazapine and 50 μM Mirtazapine, indicating a potential protective property of Mirtazapine on TJs.

### Mirtazapine increased the expression of Nrf2 in LPS-challenged Bend.3 brain endothelial cells

**Figure 7.**
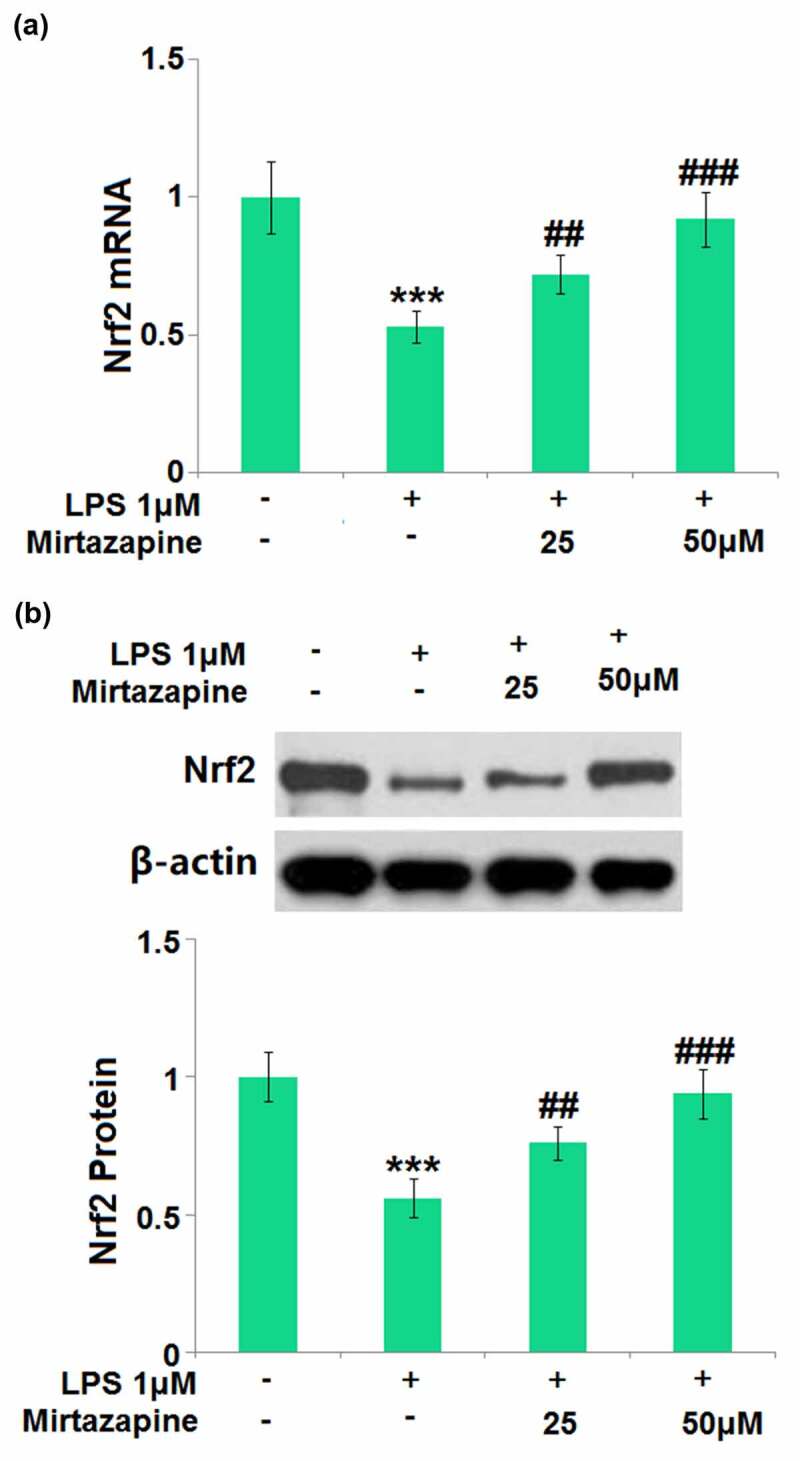
Mirtazapine increased the expression of Nrf2 in LPS- challenged Bend.3 brain endothelial cells. Cells were treated with LPS (1 μM) in the presence or absence of Mirtazapine (25, 50 μM) for 24 hours. (a). mRNA of Nrf2; (b). Protein of Nrf2 (****, P < 0.001 vs. normal mice group; ##, ###, P < 0.01, 0.005 vs. LPS treatment group).

Nrf2 is a vital transcriptional factor involved in the protection of brain endothelial integrity in the development of SAE [25]. The impact of Mirtazapine on the expression level of Nrf2 was subsequently checked. Nrf2 (Figure 7(a,b)) was found dramatically downregulated by LPS but greatly upregulated by 25 Mirtazapine and 50 μM Mirtazapine, indicating that the protective effect of Mirtazapine might be associated with the upregulation of Nrf2.

### Silencing of Nrf2 abolished the protective effects of Mirtazapine on endothelial permeability against LPS in Mirtazapine-treated cells

**Figure 8.**
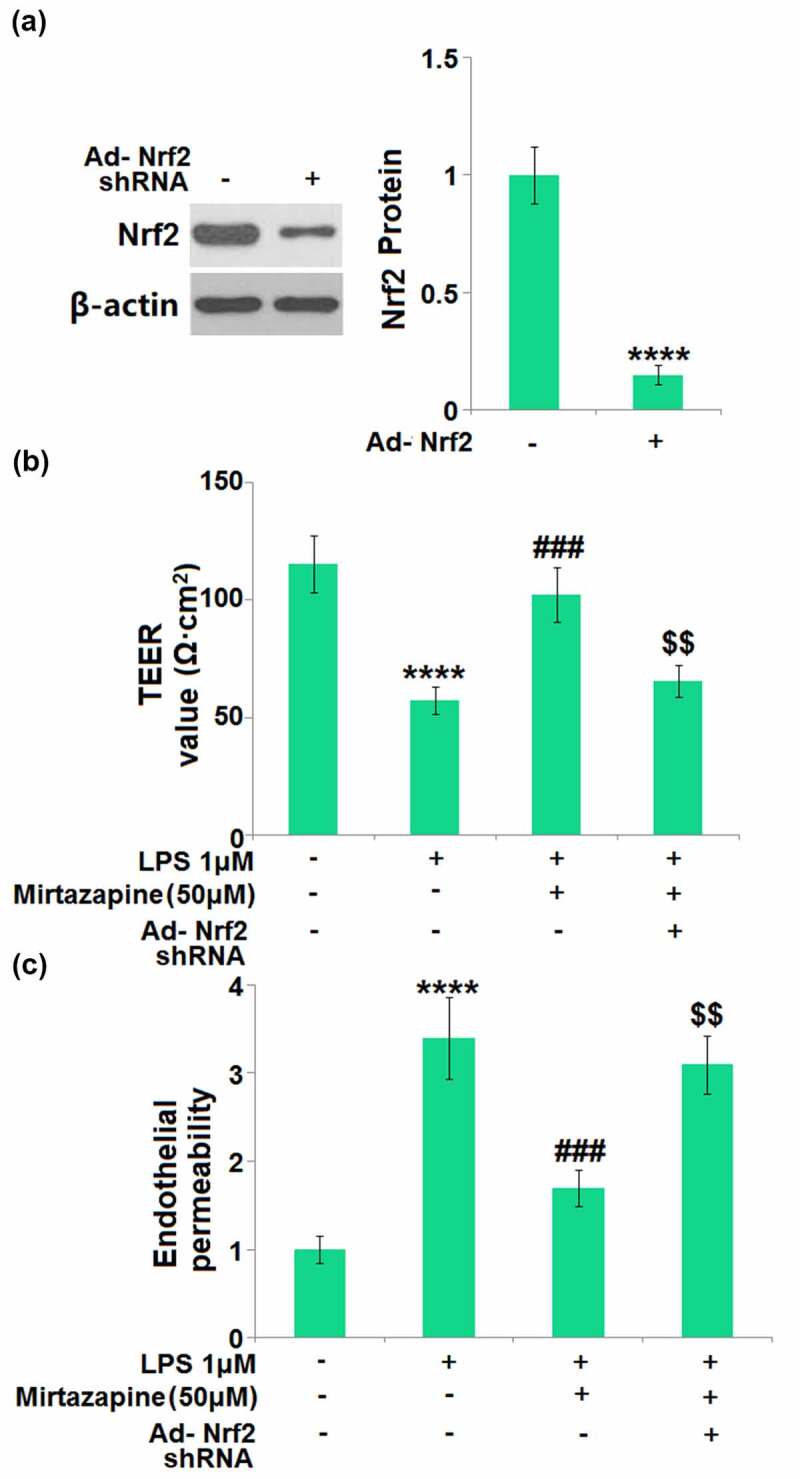
Silencing of Nrf2 abolished the protective effects of Mirtazapine in endothelial permeability against LPS. Cells were transduced with Ad-viral Nrf2 shRNA, followed by stimulation with LPS (1 μM) in the absence or presence of Mirtazapine (50 μM). (a). Western blot analysis revealed a significant knockdown of Nrf2; (b). TEER value was determined; (c). Endothelial permeability (****, P < 0.001 vs. normal mice group; ###, P < 0.005 vs. LPS treatment group; $$, P < 0.01 vs. LPS+Mirtazapine group).

To identify whether the protective effect of Mirtazapine was mediated by the upregulated Nrf2, endothelial cells were transduced with Ad-viral Nrf2 shRNA, followed by stimulation with LPS (1 μM) in the absence or presence of Mirtazapine (50 μM). Firstly, the Nrf2 knockdown efficacy was verified by Western blotting assay (Figure 8(a)). The TEER value (Figure 8(b)) was greatly declined from 115.2 Ω·cm^2^ to 57.5 Ω·cm^2^ in endothelial cells by LPS, then greatly elevated to 102.4 Ω·cm^2^ in Mirtazapine-treated cells. After the knockdown of Nrf2, the TEER value was reversed to 65.7 Ω·cm^2^. The results of FITC-dextran (Figure 8(c)) indicate that the monolayer permeability was elevated in LPS-treated cells, then greatly repressed by Mirtazapine. After the knockdown of Nrf2, the monolayer permeability was reversed significantly. These data collectively reveal that the protective effects of Mirtazapine on endothelial permeability against LPS were abolished by the knockdown of Nrf2.

## Discussion

The barrier function of the BBB is mainly maintained by the specific BMECs and TJs located among BMECs [26,27]. Endothelial cells are the first line cells in direct contact with blood and are more sensitive to the substance changes in blood circulation. Under the condition of sepsis, a series of pathological changes including activation, injury, and apoptosis are induced on BMECs. Overactivation on BMECs is induced by the stimulation of pathogens and endogenic inflammatory factors, triggering the damage and apoptosis of BMECs to disrupt the anti-inflammatory and pro-inflammatory balance, and the anticoagulation and coagulation balance. Consequently, local inflammatory reactions and microcirculation disorders are aggravated by the enhanced pro-inflammatory and coagulation progressions [28,29]. Under the state of sepsis, the activation of the monocyte-macrophage and complement systems is induced, triggering the excessive release of inflammatory mediators, such as TNF-α, IL-1, IL-8, bradykinin, and histamine. These activate the NF-κB pathway to facilitate the transcription of such genes as endothelial cell cytokines, adhesion factors, growth factors, and chemokines. As a consequence, the inflammatory reactions are aggravated to aggrandize the damages on BMECs [30]. We found that in both LPS-stimulated mice and LPS-treated endothelial cells, excessive production of inflammatory cytokines was observed, accompanied by the increased BBB and endothelial monolayer permeability. After treatment with Mirtazapine, the severe inflammation in LPS-treated animals and endothelial cells was significantly mitigated, with alleviated BBB and endothelial monolayer permeability, revealing a protective function of Mirtazapine on BBB integrity through ameliorating the injury on BMECs.

TJs are critical structures that maintain the integrity of the BBB [31]. Previous researches [32] reveal that the integrity of the BBB could be disrupted by LPS through declining the TEER of the BMECs monolayer, which is verified by the increased permeability to dextran molecules, consistent with the results observed in the present research. The normal permeability of the BBB is mainly maintained by the mutual effects of TJ proteins such as claudins and occludin, and functional proteins such as ZO-1, F-actin, and myosin [33]. When the brain endothelial permeability is increased, TJ-related proteins, including ZO-1 and occludin, are downregulated [31,33,34], which is consistent with the data observed in the present research. After treatment with Mirtazapine, brain endothelial integrity in LPS-treated mice was pronouncedly alleviated, accompanied by the upregulation of ZO-1, indicating that the protective effect of Mirtazapine on BBB disruption might be associated with the repair of TJ structure. Although we did not examine other tight junction proteins, it is likely that Mirtazapine regulates other tight junction molecules.

*In vitro* studies reveal that the oxidative damage on vascular endothelial cells can be induced by LPS via direct upregulation of the activity of NADPH oxidase and increasing the production of ROS in BMECs, contributing to the disruption of BBB integrity [35]. Nrf2 is a basic leucine zipper redox-sensitive transcription factor that regulates the activity of redox under harmful stresses [36]. Nrf2 is regularly anchored in the cytoplasm by kelch-like ECH-associated protein 1 (Keap1), a natural inhibitor of Nrf2. Under the state of oxidative stress, Nrf2 dissociates from Keap1 and is transferred into the nucleus to enhance the expression levels of multiple anti-oxidative enzymes, such as glutathione peroxidase and heme oxygenase-1, which protect cells or tissues from oxidative damages [37]. Recently, the regulatory effect of Nrf2 on TJ-related proteins and the TJ structure has been widely reported [38,39]. In this research, the reduced Nrf2 level in LPS-stimulated BMECs was greatly reversed by Mirtazapine, revealing an activating function of Mirtazapine on the Nrf2 pathway in BMECs. Additionally, the protective effects of Mirtazapine on endothelial permeability against LPS were abolished by the silencing of Nrf2, revealing that the protective function of Mirtazapine against BBB and TJ structure disruption was mediated by the upregulation of Nrf2. Mirtazapine is a potent 5-HT2A receptor antagonist. A previous study shows that the same family 5-HT1A receptor agonists promote the nuclear translocation of Nrf2 in astrocytes [40,41]. Therefore, we tested for the influence of Mirtazapine on Nrf2 in brain endothelial cells.

In future work, the regulatory effect of Mirtazapine on oxidative stress in LPS-treated BMECs will be further investigated to check whether the protective effect of Mirtazapine is associated with both the upregulated TJ proteins and the alleviated oxidative stress mediated by Nrf2.

The limitation of the current study has to be discussed. Firstly, we adopted LPS-induced SAE as an endotoxin shock model. Due to the high dose of LPS that is used to induce systemic inflammation, there are criticisms about this model. We have to emphasize the difference between rodent models and human disease. The mouse model of sepsis is relatively more resistant to endotoxin LPS, and the lethal dose in mice is much higher than that to induce sepsis in humans. Also, the timing of the development of sepsis is very different from the pathology of sepsis in humans. Most critically, the use of LPS-induced sepsis fails to predict the outcome of clinical trials of sepsis in humans [40]. Secondly, we only examined the brain endothelial cells in our study, and did not include the influence on pericytes or astrocytes. Thirdly, we used mouse cell lines bEnd.3 for easy propagation. The cell line is an immortalized cell line, which could be different from the cells in situ [22]. Ideally, the study should include primary human brain microvascular cells. Lastly, we performed the radioactive isotype-based tracing experiment to test permeability in homogenized brains, which is not the best permeability assay method.

## Conclusion

In summary, our study shows the administration of Mirtazapine significantly improves SAE-associated brain vascular permeability and inflammation in an LPS-induced mouse sepsis model. In cultured brain endothelial cells, Mirtazapine ameliorated LPS-induced endothelial hyperpermeability and inflammation. Mechanistically, the transcriptional factor Nrf2 is essential for the effect of Mirtazapine on endothelial cells. We conclude that the 5-HTA2 antagonist Mirtazapine may have a beneficial effect in a sepsis model by protecting brain endothelial cells integrity by upregulating Nrf2.

## Supplementary Material

Supplemental Material

## Data Availability

The data that support the findings of this study are available from the corresponding author upon reasonable request.
