# Supplementary figures and images for: The protective effects of Mirtazapine against lipopolysaccharide (LPS)-induced brain vascular hyperpermeability
Source: Bioengineered. 2022 Jan 26;13(2):3680–93. doi: 10.1080/21655979.2021.2024962 (PMC8973832; doi:10.1080/21655979.2021.2024962)

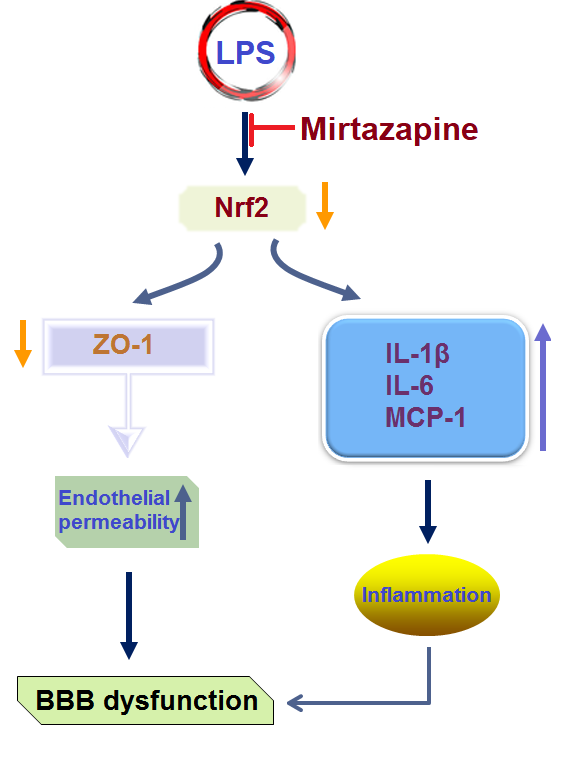

Supplement: Supplemental Material [file KBIE_A_2024962_SM9080.zip › supplementary/Graphical abstract.tif]
